# The Meaning of Becoming a Mother. A Phenomenological‐Hermeneutic Study

**DOI:** 10.1111/scs.70011

**Published:** 2025-03-16

**Authors:** Åsa Gamgam Leanderz, Margaretha Larsson, Frida Lygnegård, Caroline Bäckström, Maria Henricson

**Affiliations:** ^1^ School of Health Sciences University of Skövde Skövde Sweden; ^2^ FamCeH, School of Health Sciences University of Skövde Skövde Sweden; ^3^ Department of Rehabilitation School of Health and Welfare, Jönköping University Jönköping Sweden; ^4^ Department of Caring Science University of Borås Borås Sweden; ^5^ Jönköping Academy, School of Health and Welfare Jönköping University Jönköping Sweden

**Keywords:** becoming a mother, meaning, phenomenological hermeneutic, reflexive, transition, transition to parenthood

## Abstract

**Background and Aim:**

The transition to motherhood is a life‐changing period with dilemmas relating to meaning and relationships. These experiences are described as individual and can be related to existential questions and relationships with family and others, as well as whether healthcare professionals, when meeting with becoming mothers, miss promoting existential aspects. This study aimed to illuminate the meaning of becoming a mother during the transition to motherhood.

**Methodological Design:**

The study used an explorative design with a phenomenological hermeneutic approach. Data were collected through open‐ended interviews with 22 mothers, eight of whom were pregnant at the time of the interview.

**Results:**

The meaning of becoming a mother was experienced as being profoundly touched and changed. By a sense of belonging and being present in the moment, mothers open up to the possibility of being profoundly emotionally affected, which is understood as essential to their meaning of becoming a mother.

**Conclusion:**

This study reveals that mothers experience intense emotions that can make them vulnerable and open to change, which seems to promote their development of themselves.

## 1 Background

Being a paradoxical and pivotal life event, the transition to motherhood can be explained as an existentially changing event [1], a period with dilemmas relating to meaning, vulnerability, and relationships [1, 2, 3]. The development process of becoming a mother is explained as complex, beginning during pregnancy and continuing after the post‐partum period [4]. The characteristics of the transition are described as experiencing psychological changes, adapting to physical changes, changing the social perception from being a woman to someone's mother, redefining the relationship between family and others, adjusting priorities and forming and developing a relationship with the newborn [5]. A healthy transition to motherhood involves a sense of well‐being, comfort with the behaviours required as a mother and role achievement [6]. The maternal identity seems to be established through a commitment to and involvement in defining her new self and is described as evolving through new challenges by acquiring new skills [7]. Usually, a mother's experience of her changing self has been described within transition theory as limited to how to become a mother to the baby. Nevertheless, the mother's experience of the changing self during childbearing is highly individual [8].

Mothers, regardless of parity, can feel unprepared to navigate various experiences, such as depression, anxiety, physical recovery, breastfeeding, and infant‐care experiences [9]. As the developmental transition to becoming a mother is associated with this wide range of emotions [3], it can make mothers vulnerable [10]. The mother's vulnerability can become apparent during the stage of childbearing due to the proximity to life and death and is revealed in women's anxiety [11].

When becoming a mother today, first‐time mothers (aged 30) and second‐time mothers (aged 32) [12] live in a digital society [13]. Childbearing mothers are often increasingly drawn to social media to get support and help from other mothers and establish intimate relationships in online environments [14, 15], making them set high expectations for themselves [16]. A digitalised society, however, presents both opportunities and challenges. Research shows that digital resources can improve parents' health while providing opportunities to extend social connections, enhancing their capacity to understand and adapt to parenthood [17], helping when mothers feel isolated, but also creating or exacerbating anxiety [15].

For healthcare professionals to recognise mothers' vulnerability during the transition to motherhood, it is vital to prioritise the maintenance of mothers' health. Existentially, health can be experienced as well‐being, which describes the ability to continue engaging in the projects that matter and characterise one's life [18]. Existential, a widely used concept, encompasses different meanings; within a European context, it is described as a framework including secular, spiritual, and religious meanings [19].

From the lens of existential philosophy, existential issues are regarded as universal [20], and existentialists share a common concern for the lived experience [21]. As such, mothers are free to choose and are responsible for their actions and, through their choices, form themselves as life unfolds [21]. Realising this often leads to anguish, as mothers must confront the responsibility of making choices and come to terms with being mothers within their circumstances. Existentialists place importance on authenticity, which involves being true to oneself [21].

A coherent and authentic transition to motherhood can be enhanced by dealing with existential aspects within maternity care [22] and child healthcare. Midwives and Child Health Care (CHC) nurses meet with mothers repeatedly during their maternal transition and thus have various possibilities to support their existential issues. Research has shown that a course in existential communication for midwives increased their understanding of the importance of reflection in practice and communication about existential aspects with parents [23]. Existential aspects of the transition to parenthood are closely related to meaning in life [22]. Meaning is individual and can be related to existential questions [24]. In meaningfulness lies parenthood's potential rewards rather than happiness [25]. Earlier research reports a significant lack of understanding regarding the professional's role in addressing existential needs, with values taking precedence over this aspect of care [26]. Ignoring existential aspects of childbirth can negatively affect a mother's psychological, emotional, and physical well‐being [24].

Given the multitude of experiences and challenges that pregnant and new mothers encounter in today's society and that previous research highlights vulnerability during the transition from pregnancy, childbirth, and into parenthood, it is important to address what it means to become a mother to gain an understanding of what matters to mothers. This understanding can be important for healthcare professionals to form individual support for a healthy transition to parenthood. Therefore, this study aimed to illuminate the meaning of becoming a mother.

## 2 Research Design and Methods

This qualitative study uses a phenomenological hermeneutic approach, as outlined by Lindseth and Norberg [27]. The research illuminates the meaning of becoming a mother during the transition to motherhood. The study is guided by the consolidated criteria for reporting qualitative research (COREQ) checklist [28].

**FIGURE 1:**
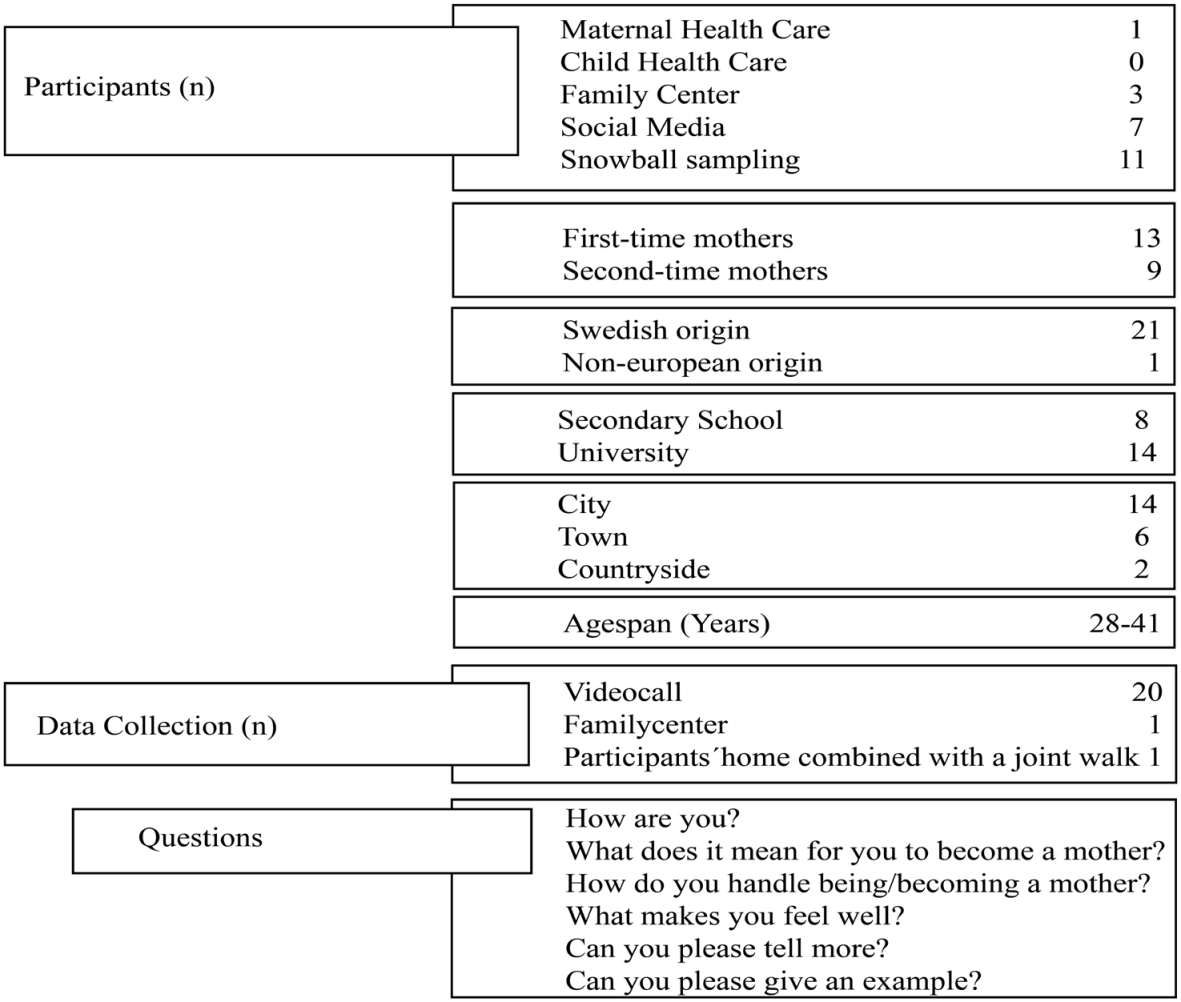
Participants and data collection.

The study was conducted from August until December 2022 in Sweden. Recruitment for this study took place via Maternal Health Care, Family centres, social media, and snowball sampling [29] (see Figure 1) from various regions across Sweden. The inclusion criteria were pregnant mothers (starting from gestational week 25) or new mothers who had given birth to their child in the last 2 years. The developmental transition to becoming a mother, starting during pregnancy, is associated with a wide range of emotions [6] and completing the psychosocial transition may take months or years [30]. The exclusion criteria were mothers younger than 18 years old and those undergoing psychiatric treatment. All included participants were in a heterosexual relationship with the partner with whom they had their child. All but one of the subjects lived in Sweden when the study was conducted.

Open‐ended [27, 31] interviews with 22 mothers, first‐time (*n* = 13) and second‐time (*n* = 9), were conducted at various stages, with one interview with each participant, starting from pregnancy (week 25) (*n* = 8) and continuing until 16 months after giving birth (*n* = 14). Open‐ended interviews allow a rich, deep, and textured picture to be created in the conversation between the interviewer and the interviewee through initiating and follow‐up questions [32]. The first author made the interview guide based on previous research [33] and the study's aim. The interview guide was pilot‐tested in one interview, and after this interview, questions were adjusted. All interviews were conducted, audio recorded, and transcribed verbatim by the first author. The interviews lasted between 37 and 75 min.

The data were analysed using the phenomenological hermeneutic approach described by Lindseth and Norberg [27, 31]. After a first reading of the data to become familiar with the text, a naïve understanding was formulated whereby the immediate view of the text is based on the preunderstanding [34]. The first author's preunderstanding stems from being both a midwife and a mother, which shaped the interpretation process. Recognising that awareness only captures aspects of this preunderstanding, critical reflection was essential during the analysis. This reflection was revised, broadened, and deepened through the author's awareness. When the preunderstanding proved too superficial during text interpretation, it was refined through studying relevant literature and discussions with the co‐authors [27] from different healthcare disciplines (midwives, occupational therapist, primary health‐, or intensive care nurses).

Ricoeur [34] stated that the subsequent structural analysis validated the naïve understanding. The structural analysis began by dividing the text into meaning units. The condensed meanings of the text were reread, reflected on regarding similarities and differences, and regrouped several times into subthemes, after which they were condensed and abstracted to form themes and a main theme. To validate the themes, they were compared with the naïve understanding. In this phase, questions about the text that focused on the meaning of becoming a mother were asked. There was an effort to view the text as objectively as possible. This means forgetting about the participants, focusing on the words, and allowing the meaning to appear. This was achieved by decontextualising the meaning units from the text as a whole. The meaning units were considered independent from the original text's context. Therefore, the meaning units needed to be long enough to contain one essential meaning. When several meanings occurred in the meaning units, they were further divided. By this phenomenological hermeneutic method [27], there was a dialectic movement between understanding and explanation; thus, the condensed meaning was reread, reflected on regarding similarities and differences, and regrouped several times. Text interpretation means entering the hermeneutic circle, repeatedly going from parts to form a new whole [27]. This process involved recurrent discussions with the co‐authors, after which the analysis resulted in one main theme, two themes and six subthemes. Quotations are used to validate the trustworthiness of the analysis.

### 2.1 Ethical Considerations

Ethical approval was received from the Swedish Ethical Review Authority (Dnr: 2022‐00413‐01; Dnr: 2022‐02868‐02). The professionals at the caring units supplied written study descriptions, while the first author distributed them during snowball sampling. These descriptions included information about the study's aim, procedures, inclusion and exclusion criteria, and their right to withdraw their participation without explanation. The respondents gave written consent to participate digitally before the interviews [35]. The data were stored, secured, and managed during the study according to the General Data Protection Regulation (GDPR) and national law [36].

## 3 Results

### 3.1 Naïve Understanding

For mothers, the meaning of becoming a mother during their transition to motherhood means feeling reverence for how a child can be created and later marvelling at their child's development. It also means reflecting on having created eternal bonds, new meaning, gratitude, hope and direction in life. Becoming a mother relates to feeling new emotions, being directly touched in one's inner self by daily life situations, and recognising one's vulnerability. They seek confirmation and help from friends, family, and other parents with similar experiences. Becoming a mother during the transition to motherhood is also about seizing the moment and being open to feeling love and feeling loved by the child, the partner, and the larger family. Becoming a mother means an increased reflection on what is essential and letting a new focus on life emerge.

### 3.2 To Be Profoundly Touched and Changed

Becoming a mother means being profoundly touched within and opening up to change and new dimensions of life during the transition to motherhood. Present in the moment with their entire being, their surroundings touch them, and the love for their child, other people, and nature makes them change. Being open to change in the transition is essential as the transition into motherhood is described as inevitable and all‐encompassing. Their change entails their increased bodily awareness and the development of their new self. A mother's change can also be seen in heightened emotions and becoming more vulnerable and sensitive to the surroundings, such as becoming sensitised to possible dangers for their children. Mothers actively seek supportive communities where they feel a sense of belonging, and they consider what they receive and offer to those around them. It describes a journey in the present, with recurrent reflections connecting the present to the past.

This main theme is further explored in two themes and six subthemes (see Table 1).

**TABLE 1:** Results for the main theme, themes and sub‐themes.

**To be profoundly touched and changed**	
Sense of belonging	To balance being part of different contexts and managing on one's own
	To be open to love
	To be mothered
To be present with their whole being	Deepened bodily awareness
	Inherent vulnerability
	To develop their new self

#### 3.2.1 Sense of Belonging

A sense of belonging seems to be an essential meaning when becoming a mother, and it relates to being part of a community whose members also have experience with parenthood. Even if their experiences differ, the mothers describe a sense of belonging that addresses the emotional aspects of being a mother. To know that others have managed to become mothers before them relates to a sense of belonging. The unconditional love towards their children profoundly touches the mothers. The need for caring relationships becomes apparent to mothers during pregnancy. Consequently, most need to socialise with others and be cared for by them to feel well.

#### 3.2.2 To Balance Being Part of Different Contexts and Managing on One's Own

Mothers balance being part of different contexts and managing on one's own. This means acknowledging the importance of creating emotional space within one's self. Simultaneously, it involves being open to assistance from others in various settings, including family and friends. They change their focus to an important, limited group of people. It is described as important to be alone in a small family, to create a personal bubble, and to bond through breastfeeding. As one mother narrates:

I have been thinking a bit about how it is that this is what motherhood is all about, that I know what to do if I just listen to myself and not many others, and it usually turns out well. (208)

Mothers can reflect that children have been born throughout the ages and that others have managed it before them. Some mothers realise that existence with children does not always mean ‘to be up in the clouds’– it can also be overwhelming, related to extreme tiredness, which can affect the mother's understanding of the situation. The mothers describe that their well‐being is positively affected when daily chores flow smoothly, and they can be touched by the help of others, including their parents and siblings, as well as in communities such as an open preschool and church. Meeting friends with children is prioritised, and other's individual experiences can be discussed, which can form support. The mothers search for belonging through digital media, such as Facebook groups, to manage themselves and seek approval and affirmation to exchange experiences with like‐minded people who undergo the same thing, to participate in forum discussions and ask questions. They seek information from digital media but must also navigate and sift through it to ensure its accuracy and relevance.

Overall, the mothers want reciprocal relationships. Successful relationships require both partners to be responsive to each other's needs. Daily life requires more work than before. It is also about showing interest in their partner's day, sharing chores, laughing together, wanting to make each other happy, and having mutual plans. One mother narrates:

But, at the same time, you know that there is some light in the tunnel when we will have time for each other again (laughter). (107)

The mothers feel their partner's presence, even if it is described as challenging work to maintain their relationship.

#### 3.2.3 To Be Open to Love

Love describes reflecting an inner warmth. The mothers experience a natural, protective, and unconditional love towards their children that profoundly touches them. They feel a natural need to give their children love, even before birth. Nevertheless, they are unprepared for the power of their love for their child and undergo an emotional change. This change can happen quickly, though it often takes time. As mothers enter motherhood, some realise they might not have initial feelings for their child, experiencing the child as a stranger who is suddenly with them all the time, though love would emerge and grow within them over time. This type of love is described as different, more tangible, and exists on another level than any love they have known. Becoming a mother compares to falling in love; however, it reflects as much stronger than love for their partner. A profound connection can also be felt when the mothers feel God's presence; they experience it as veritable, not just a feeling. Strong feelings such as these also raise concerns about whether they can be open to love:

Should I take care of another person? Should I love another person? It feels so vulnerable and scary. Well, should I be loved by someone else? It is a bit like a love relationship. Am I such to be loved to love someone? What is it, really? (213)

Mothers experience an extended circle of love as they share a mutual, unifying joy and interest in their new child with their partner, parents, and siblings. They realise that loving and feeling loved back indeed means something in life. The openness for love replaces anxiety about other people's opinions, frivolity, and uncontrollable anger. They feel humility, increased compassion, and a greater understanding of unwell children and parents who struggle with everyday life, ultimately making them more caring and less selfish.

#### 3.2.4 To Be Mothered

They reflect on being seen as themselves, as a person, and knowing that their mother kindly cares for them and is available, strengthening their well‐being. A close relationship with one's mother can change and deepen during pregnancy and motherhood. Their mothers know their family well and provide support when their partner is absent. Sharing the experience of being a mother becomes a natural basis, which results in moments where the mother can herself be a child again:

This is what I need; I want to be mothered. Now, she calls often. How is my daughter? And it is so very nice, not what about the baby like everyone else writes, but what about you? (213)

The experience of feeling unseen occurs when mothers and other close family members give all their attention to the child. They describe a deep frustration of feeling invisible when the child receives the focus they had before from family and friends. When the focus is mainly on the child, mothers can communicate clearly with their mothers that they want to be the focus, to be mothered, heard, and held as if holding their child. When being mothered, they feel enabled to take care of their child:

I take care of the child…then I need someone to take care of me so I can take care of the child. (213)

Having mothers far away or not in their lives affects the mothers' transition, making everyday life more difficult. Simultaneously, mothers not mothered by their own mothers can seek support and have a safety net in the form of other women who have experience with being mothers—for example, in‐law parents. Mothers without a safety net can feel guilt; for example, when an older child needs to be left with someone that the child does not know when the mothers have meetings within healthcare.

#### 3.2.5 To Be Present With Their Whole Being

To be present with their whole being means reflecting on and being present at the moment with a focus on the family's well‐being, which describes a central meaning of becoming a mother. Insights into their body's abilities deepen and change their bodily awareness. Mothers change by experiencing inherent vulnerability, which relates to more intense, newer feelings, increased sensitivity, and closeness to their feelings. Seeing their child's defenceless gaze makes the mothers feel more present, and recognising their total dependence on them for their existence profoundly affects them and urges them to develop their new selves.

#### 3.2.6 Deepened Bodily Awareness

Being pregnant means a deepened bodily awareness that is both concrete and abstract for the mothers. Concretely, it means that mothers can feel distressed and uncomfortable with their changed physical bodies during pregnancy:

It was focused on the body …that is when it started …then it had to disappear completely as soon as I get her out for me; it became a bit of a mental ghost, then we have to hurry, like it was really about yourself, not yourself but what everyone else thought.(205)

They reflect on physical closeness to their child during pregnancy, in their body as total and never recurring. The mothers are profoundly amazed and impressed by their bodies' complexity, which enables the child to develop from an embryo into a fully functional human. The feeling of being in a flow—that the mother and her body work together with her child and its body within her, as a team, up to and during childbirth—conveys a deepened bodily awareness. As one mother describes it:

We had a home birth with [the baby's name] here. I think it can contribute to my feeling now being as good as it is that we got off to a good start, became safe at home and just got to cuddle (208)

After childbirth, the mothers can experience their bodies taking care of everything. For example, producing breast milk and breastfeeding stimulate their child's growth and promote mutual well‐being. On the other hand, mothers can experience that they only mean food to their children. They also feel distressed about their changed physical bodies, experiencing discomfort, with some women even avoiding going outdoors after childbirth:

When you are going to meet people from the outside, you have to look like before a little quickly. (205)

The mothers feel that constantly being physically near their children during the day satisfies their need for bodily closeness. When their child is asleep, mothers reflect that closeness grows in their relationship with their partner by sitting together on the same sofa, even when not physically in contact.

#### 3.2.7 Inherent Vulnerability

The mothers change profoundly from experiencing inherent vulnerability relating to more substantial and newer feelings, which refer to increased sensitivity, the tendency to cry, and worries about possible accidents happening. Mothers are closer to their feelings and experience varying degrees of patience. They become more sensitive to their partners' tone of voice and, for example, have difficulty handling sarcasm from family and friends. One mother describes her emotions related to healing metaphorically as a big wound related to the placental release during childbirth that takes time to feel whole again:

A big wound that no one sees, but is huge and needs to heal, but takes a long time, so for me, it is just very emotional, that it has gone like this fast that it has gone from zero to a thousand. (213)

The inherent vulnerability can relate to the fear of becoming a burden in their relationship, showing themselves crying. Their inherent vulnerability is reflected by the will to protect their child. For example, the instinct to run away with their child from encounters with strangers during walks. This relates to experiencing the need to be on their guard against unseen environmental threats. It can manifest as thoughts of disaster or fears of hurting their child accidentally by falling, dropping a knife on them, or falling asleep on them:

I can imagine that God, someone will take the pram and run away. [I]f I carry her in a carrier, I am terrified that I will fall. [I]f I manage to get some sleep with her in my arms, then I am afraid she will suffocate. (205)

One's inherent vulnerability during pregnancy can be interpreted as fear of missing out if the baby is not feeling well in the womb, related to the mother's inability to see her child. The mothers can be anxiety‐ridden about the intangible; they can be reassured that the infant is alive by the child's movements and the partner's questions about their child's movements, for example.

#### 3.2.8 To Develop Their New Self

To develop their new self means that the mothers feel calm, care for themselves, change, and develop as human beings through their new self. The eye contact with their child touches them profoundly and awakens a deep sense of responsibility. Nonetheless, emotionally and spiritually, understanding what it means to develop their new self as a mother can be a long process. The mothers reflect on who they are in their new identity and perceive themselves and their surroundings in a new way. The mothers need to be confirmed as a person and, as mothers:

Yeah, but that is the thing about getting a new identity, showing, well, who I am now? Then you have known yourself all your life. You know a little about who you are. I have not really connected with the mother, being a mother. It has been scary for me, that part. I still think it is kind of scary. Actually, there is something very deep there. (213)

The new focus on their children can consume their whole existence. Reflecting on themselves, mothers can develop their new selves by focusing on their children, following their instincts, and doing what feels right. Mothers are careful to spend time communicating with their children rather than using social media. However, using their phone while their child is awake gives some mothers a guilty conscience. The mothers can feel a split in themselves when they experience limited freedom, and life can no longer be lived as before. To feel whole, they have to make plans and have projects. They have to recognise that life has meaning beyond becoming a mother. Spending time alone and with friends brings about change and creates life space:

To talk outside of myself with another adult person (laughter) without being interrupted, adult socialising, to socialise with peers and not just with my children. (214)

The mothers gain a renewed perspective on life. They find their vitality again when they can relax, spend time in nature, devote time to physical exercise, and engage in self‐reflection by reading poetry, writing, and listening to or creating music.

## 4 Discussion

The main result of this study indicates that to be profoundly touched, that is, being deeply affected and changed, describes the meaning of becoming a mother. Pregnancy and childbirth seem to be profound experiences with significant existential meaning for the mothers and their families [37, 38]. Mothers experience a sense of belonging, and similarly, a recent review revealed that the woman's place in her social world and inner self was identified in terms of the meaning and understanding of maternal well‐being [39]. Most of the interviewed mothers stated that being entirely present was an experience that could make them profoundly touched. In other words, they experience new and more intense emotions when subjectively present. Being present with their whole being is essential since, according to Meleis [10], engagement is needed for a transition to start, which is defined as the degree of involvement in the process. Subsequently, mothers realised that loving and feeling loved truly meant something in life. Allowing oneself to be profoundly emotionally affected is described as being true to oneself, which belongs to the authenticity central to existential understanding [21]. Our results illustrate that mothers realise this transition to be inevitable, all‐encompassing, and overwhelming, and becoming a mother is described as a major life transition that can feel overwhelming [1].

When the mothers experience a flow in their everyday chores, they reflect on that as a central aspect of the meaning of becoming a mother. According to Meleis et al. [40], movement and flow over time characterise transitions. ‘Going with the flow’ is described as one coping strategy that first‐time mothers adopt during pregnancy as they await the unknown event of childbirth [41]. Moreover, according to Bornemark [42], there is no need to immediately find effective answers since an existential approach entails daring to face uncertainty, pain and change [42].

The transition changed and personally developed the mothers in our study through their new selves. In earlier research, this maternal personal growth is described as self‐discovery [43] but can also be accompanied by a feeling of losing their former identity from a previous life [16]. Previous research highlights that maternal self‐esteem, competence and autonomy matter in order to flourish in their new, integrated identity as a ‘woman and mother’ [44]. In our results, mothers reflect upon their experiences, which can be seen as a way to gain new [31] and deeper understanding of self and others [24]. Reflecting can handle questioning life conditions and dilemmas, which can support recovering the balance and getting in touch with one's driving force –meaningfulness [45].

Our results indicate an inherent vulnerability related to more intense and newer feelings, such as fear of missing out if the baby is not feeling well in the womb. Correspondingly, a recent study explored awareness of death as experienced in the parenthood transition and found two overarching themes: the fragility of our loved ones and awareness of my own finitude [46]. Corroborating Meleis et al. [10], pregnancy and motherhood transitions may lead to heightened vulnerability. The vulnerability can appear in women's worries [11]. According to Sigurdson [47], an individual's existential health is defined as a reflexive experience of health and in its approach to suffering; health is achieved by learning how to suffer [48]. To clarify, Sigurdson believes suffering involves taking a proactive, intentional stance towards painful psychological or bodily realities. Hence, suffering is described as embedded in existential health. For example, one mother reflects metaphorically in our results concerning the placental release leaving a wound, meaning individual experiences that demand time to heal.

Mothers want to be mothered, strengthening their well‐being in the present study. Our results show that motherhood can deepen a mother's relationship with her mother, particularly regarding their now‐shared experience with pregnancy and motherhood. Likewise, previous research has outlined the importance of the mother–daughter relationship, especially regarding practical postnatal support [49].

According to our results, a sense of belonging is essential to the meaning of becoming a mother, which relates to being part of a community whose members also have experienced parenthood. Towards this end, the mothers in our study connect online and offline in various settings. Earlier research has shown that mothers mainly engage in social media to find relevant information, establish social connections with other mothers, and read about people in the same situation as themselves [15, 50]. This engagement aligns with our results, showing that mothers can feel a sense of belonging when people in the same situation confirm their experiences. They also actively contribute content to social media groups [15].

Moreover, most of the interviewed mothers stated being entirely present when, rather than using social media, spending time communicating with their children to get to know them with eyes and words. This can be related to a sense of experiencing tranquillity when making space for and allowing things to exist as they are [51]. The eye contact with their children touches mothers and awakens a deep sense of responsibility, making them more available to their children. When mothers profoundly connect to and become absorbed in the moment, they tune into a focus offering oneness [52]. Mother‐to‐child bonding and support should receive more attention when midwives address the transition to parenthood during prenatal visits [53]. Mothers themselves emphasise that respectful, compassionate care transcends simple politeness and is essential to childbirth [54]. Compassion can be a power source for caring, constituting a fundamental mood of reciprocity and presence [55]. For consideration in one's professional and private life, genuine care can be, for example, eye contact and a kind touch, and is about emotional availability [55].

Our results show that most of the mothers stated being entirely present and performing recurrent reflections, making a journey that connects the present to the past, which can also be viewed from the dwelling dimension of the existential theory of well‐being. That is, they are grounded in the present moment, supported by the past, and they are open to a future that is calling [51]. The mobility dimension of the existential theory of well‐being, as mothers emphasise the call of the future and the energetic feeling of possibility [51], is described by mothers as gaining a new perspective on life related to finding their vitality again.

## 5 Strengths and Limitations of the Study

A strength of the study was that 22 mothers participated, contributing to a thicker description that enhanced the transferability of the findings [56]. The majority of participants were found by snowball sampling (*n* = 11) and social media (*n* = 7), which might lead to a sample with decreased transferability, a study limitation. The participants, both pregnant and new mothers, contributed different perspectives, which were applied to get an overall picture of the transition process. Although being a second‐time mother, there is a transition, becoming a mother of two. It is a period that is fast for a new mother, but it can appear as a long time for pregnant women. These different perspectives enabled the entire transition process to be grasped and enhanced understanding with dense, meaning‐rich accounts of details [29]. The thick description not only enhances the transferability of the findings but also provides insight into the experience of well‐educated Swedish parents in heterosexual relationships, a relatively homogeneous group. A limitation is that no lesbian or single mothers and only one foreign‐born mother participated, which can impact the study's transferability to a contemporary, modern Western society. Most of the interviews were digital, and the pros with video were that the mothers could feel at ease in their home environment. However, the participant might find revealing their thoughts on a screen difficult. From the mother's willingness and openness in sharing their thoughts, these videos through screens might have helped them focus on themselves, not forcing eye contact, enhancing credibility.

## 6 Reflexivity

The analytical steps were carefully followed according to the method developed by Lindseth and Norberg [27, 31], strengthening the data's credibility [29] and the study's rigour. The phenomenological hermeneutic approach allows for being open to the phenomenon and a deepened understanding. Questions about the text that targeted the meaning of becoming a mother were asked. Among the initial questions was ‘What makes you feel well?’ which may have skewed the results towards positive experiences and a smooth transition. The findings might be considered as idyllic, not in accordance with previous research. This current study delves into the meaning of becoming a mother. These new findings are novel and have not appeared in previous research. However, the repeated open‐ended questions following each posed question enabled stories about what constituted a mother's hardships when becoming a mother. These hardships can be considered as insights into, for example, a vulnerability in the parents during the transition to parenthood. Quotations were employed to validate the results and strengthen their trustworthiness. The first author analysed the data through recurrent meetings with the co‐authors with broad experience in the data collection methods, which enabled practising reflexivity and strengthened the trustworthiness of the findings alongside a naïve understanding that was reformulated several times and verified through a new structural analysis to ensure coherence between the parts and the whole. The first and last authors worked closely on the analysis.

## 7 Conclusions

The present study indicates that the surrounding family, friends, and other significant persons are important to facilitate becoming a mother. This study reveals that mothers experience intense emotions that can make them vulnerable and open up to change, which seems to promote the development of themselves. By reflecting on themselves and their surroundings, they can make a journey to bring the past into the present to be themselves as mothers.

## 8 Clinical Implications

Health organisations should seek mothers on digital media to interact with them rather than merely launching read‐only information portals and expecting mothers to visit them [57]. This can be done by moderating parental forums online [58]. There is a need to promote spaces for mothers to converse with one another. Previous research suggests asking: How do you understand your role in childbirth? What does it mean to you to become a parent? [33]. Additional questions can be posed: These may include inquiries such as what it means for you to be pregnant? How do you handle unpredictability?

In the future, multiple interviews with mothers over time could offer a richer understanding of the transition. It may be interesting to conduct research that relates to existential health and the meaningfulness of becoming a parent in relation to the repeated contact with professionals in healthcare during the transition to parenthood. It is important to educate healthcare professionals in asking about the meaning of becoming a parent since the period entails a high existential meaning.

## Author Contributions

Å.G.L. completed the data analysis with M.H., M.L., C.B., and F.L. support. All authors gave input for developing the themes. Å.G.L. drafted the manuscript, and all authors, M.H., M.L., C.B., and F.L., contributed to its content and revision. All authors read and approved the final version of the manuscript.

## Conflicts of Interest

The authors declare no conflicts of interest.

## Data Availability

The data that support the findings of this study are available from the corresponding author upon reasonable request.
